# ‘Don't Assume, Ask’: A Collaboration With Consumers, Interpreters, Clinicians and Health Service Staff to Increase Video Telehealth in Culturally and Linguistically Diverse Groups

**DOI:** 10.1111/hex.70232

**Published:** 2025-03-15

**Authors:** Jaimon T. Kelly, Monica L. Taylor, Victor M. Gallegos‐Rejas, Susan Pager, Soraia de Camargo Catapan, Karen Lucas, Angel Bogicevic, Anthony C. Smith, Centaine Snoswell, Helen M. Haydon, Emma E. Thomas

**Affiliations:** ^1^ Centre for Online Health, Faculty of Health, Medicine and Behavioural Sciences The University of Queensland Brisbane Queensland Australia; ^2^ Centre for Health Services Research, Faculty of Health, Medicine and Behavioural Sciences, The University of Queensland Brisbane Queensland Australia; ^3^ Health Equity Team Women's Health and Equality Queensland Brisbane Queensland Australia; ^4^ Digital Health & Informatics, Metro South Health Brisbane Queensland Australia; ^5^ Disability and Multicultural Health Systems Policy Branch, Strategy, Policy and Reform Division, Queensland Health Brisbane Queensland Australia; ^6^ Odense University Hospital Odense Denmark; ^7^ University of Southern Denmark Odense Denmark

**Keywords:** access, culturally and linguistically diverse, equity, telehealth

## Abstract

**Objective:**

We aimed to understand gaps in telehealth use across culturally and linguistically diverse (CALD) populations in a metropolitan Australian setting and elicit solutions to support inclusive telehealth‐delivered care.

**Methods:**

Three workshops (one in‐person, two online) were conducted with purposefully recruited consumers (*N* = 6, including 2 who were also interpreters; representing six different language and cultural backgrounds) and clinical and nonclinical staff (*N* = 14) between July and October 2022. Audio‐recordings and notes were analysed using inductive thematic analysis to identify barriers and potential solutions for including CALD consumers in telehealth appointments.

**Results:**

A central identified theme was “Don't assume, ask”. CALD consumers felt they were not offered the choice for telehealth due to staff assumptions about their insufficient digital literacy, technological capability, and English proficiency. CALD consumers communicated willingness to experience telehealth benefits such as avoiding travel, and a desire to have the choice with care modality. Staff required increased training around booking a video interpreter and emphasised the role of the health service in supporting telehealth and upskilling consumers and staff. Interpreters self‐identified as “communication specialists” but felt their skills were undervalued and underutilised by clinicians. A key sentiment from CALD consumers was that they did not have equal opportunities to access healthcare overall – encompassing all aspects of care regardless of modality. Key messages for consumers, clinicians, interpreters, and executives were compiled into recommendations.

**Conclusion:**

Our collaboration highlighted the need for more education and reinforcement to promote equitable and efficient processes for accessing telehealth appointments for CALD consumers requiring an interpreter.

**Patient or Public Contribution:**

Patients, caregivers, and interpreters from culturally and linguistic diverse backgrounds were workshop participants and co‐developers of solutions to address telehealth access gaps. Final research outputs were also circulated to participants for feedback before being disseminated.

## 1 Introduction

Telehealth aims to improve equity of access to health services. Historically, telehealth has overcome geographical barriers to serve patients living in rural and remote locations [1]. Despite telehealth benefits and its post‐pandemic surge in metropolitan areas [2], certain groups of the population such as cultural and linguistically diverse (CALD) communities, are frequently not provided with telehealth options [3, 4]. This uneven distribution of benefits derived from telehealth implementation is part of a larger systemic and complex phenomenon named the “digital divide” and directly affects health outcomes [3].

The digital divide refers to the disparities between those with the resources and abilities to fully benefit from telehealth technologies, and those who face barriers to obtaining the benefits [3, 4, 5]. The digital divide disproportionally [6] affects CALD communities, a group with historically unmet health needs [3].

Our prior work identified that within a metropolitan hospital health service in Queensland (Australia), individuals requiring interpreter services were 66% less likely to use telehealth services, compared to English‐speaking Australians [7]. We have also identified, using nationally representative data, that while telehealth use differs between CALD consumers and their counterparts, acceptability and trust of these services do not, representing an opportunity to expand telehealth use within this cohort [8]. As such, it is important to understand the needs and preferences from CALD communities to provide the necessary support for digital care options like telehealth. We do not know the nuances in these access gaps, experiences and expectations of the key stakeholders involved in the provision, administration and receiving of telehealth services for CALD populations. What is particularly important to understand and explain is the specific needs and expectations of CALD consumers and interpreters, which is an evidence‐practice gap for patient‐centred care providers in Australia.

Therefore, the aim of this study was to understand identified gaps in telehealth use and access among CALD communities from the perspective of consumers, interpreters, and clinicians, and codevelop ideas for solutions to reduce these gaps.

## 2 Methods

### 2.1 Study Design

This qualitative study collected data via three workshops. Workshop 1 included CALD consumers, two of whom also worked as interpreters, and as independent consultants. Workshops 2 and 3 included health professionals and administration staff. We chose this method to maximise engagement and communication. We also wanted to allow data to be collected directly from participant responses to questions and scenarios, as well as participant discussions as a group. We involved CALD consumers and stakeholders in this project, acknowledging the importance of their high level collaboration and autonomy in the decision making process [9]. This project received ethical approval from both our university and health service Human Research Ethics Committees.

### 2.2 Setting and Context

This project took place across a metropolitan health service (Metro South Health) in Queensland, Australia. Metro South Health is the most culturally diverse health service catchment in Queensland, with more than 20% of the population using a language other than English at home [10]. Compared to the rest of the community, this group have significantly poorer quality of life outcomes, lower levels of participation in society, reduced access to services and higher social exclusion and discrimination [4, 11].

The Metro South Health Health Equity and Access Unit (HEAU) is a unique service committed to ensuring care provided in the region is equitable, accessible, and culturally safe. This project is the result of a partnership between university‐based digital health researchers, the HEAU and Metro South Health's Telehealth Team.

### 2.3 Participants and Recruitment

Potential CALD consumers and interpreters were identified by HEAU using their health service records and directly contacted via phone (using interpreters as required). The Metro South Health Telehealth Team contributed to the recruitment of clinical staff by using a snowballing sampling technique and sending out invitations via e‐mail to clinicians from identified areas of high telehealth use. Purposeful sampling of consumers ensured recruited participants had a range of telehealth experience and covered multiple ethnic backgrounds to ensure diversity, however, selection was not based on specific cultural groups. Participants provided either written or verbal consent to take part and were reimbursed a grocery store voucher worth AUD$50 for their time; we also reimbursed public transport or local parking costs. As per governance agreements, health service staff were not remunerated for their time as they took part within their employed roles during normal work hours.

### 2.4 Data Collection (Workshops)

Three workshops were conducted between July and October 2022 using group think principles [12] to explore opinions, barriers, opportunities and to codevelop recommendations for the health service. Workshop 1 was conducted in‐person at a local health service with CALD consumers, interpreters, and HEAU advocates. Workshops 2 and 3 were conducted online (via Microsoft Teams) with health service staff and clinicians. The online format was chosen due to limited staff time and their locations across many sites.

Workshops were facilitated by an experienced researcher from The University of Queensland's Centre for Online Health and supported by a Senior Telehealth Coordinator at Metro South Health and a staff member from HEAU. A variety of educational resources were used in different formats (e.g., visual, auditory, interactive) to cater to diverse learning styles, preferences, needs, language and digital literacy levels.

An agenda and priority discussion topics for each workshop were drafted by the research team and feedback was received online by academics and health administrators before finalising the material for each workshop. These topics covered: i) verbal consent and introductions; ii) telehealth definitions – and our focus on video‐consultations; iii) understanding telehealth and its purpose; iv) understanding who is missing out and who is not; v) participant experience or opinion of telehealth; vi) telehealth stories and case studies; vii) group discussions to address the gaps, key priorities each person has; and viii) co‐developing solutions to recommend the health service implement to address these needs.

The facilitator guided the conversation according to these key questions. A second facilitator moderated the chat and in‐person discussions.

Participants contributed to data collection via speaking freely or writing down their opinions on paper (in‐person workshop) or using the written chat function (online workshop). The workshop discussions were audio‐recorded. Comprehensive field notes were taken, both during and following the workshops upon reviewing each recording, to augment all data in both in‐person and online settings. Field notes specifically targeted points raised by workshop participants as key gaps in knowledge and/or actionable suggestions for better including CALD groups in telehealth appointments. At the conclusion of the workshops, the authors agreed that we had sufficient information power [13] from our sample of participants. We acknowledge that we are drawing experience from a group who access care 66% less than English speaking consumers [7], and although our sample size is small, thematic saturation was not appropriate for this sample [13].

#### 2.4.1 Data Analysis

Data were analysed using a qualitative descriptive analysis involving inductive thematic analysis [14]. After familiarisation with the data, one investigator used a two‐step process to 1) openly code the data to identify, develop categories and subcategories within the data; and 2) map each of the categories to one of four stakeholder groups: consumers, interpreters, clinicians and health service staff, and executives. A second investigator then reviewed and confirmed the categories and mapping that were relevant to the research aims and stakeholder categories. A third and fourth investigator reviewed the final classification and provided wording/phrasing adjustments and commented on the categorisation/mapping suitability. Verbatim quotes were used to represent the final categories and explain the data from the perspective of participants. Microsoft Word [15] was used to facilitate data management (tables) and basic content analysis (comments relating to attributes demonstrating feasibility and acceptability) of data.

## 3 Results

### 3.1 Workshop Timing and Participants

The consumer and interpreter workshop (Workshop 1) duration was 2.5 hours and the two online workshops (Workshops 2 and 3) for clinicians and health service staff were 1 hour each. A detailed description on workshop participants is provided in Table 1.

**Table 1:** Description of workshop participants.

Participants	Descriptions		
**Consumers (*n*** = **4)**	**Gender ratio** 1:3 male:female	**Languages represented:** Serbian Cantonese Tongan Hindi Hmong Tok Pisin Creole	
**Interpreters (*n*** = **2)**	**Gender ratio** 1:1 male:female		
**Clinical and health service staff (*n*** = **14)**	**Work site (*n*):** Tertiary hospital (4) Community health centre (6) Smaller public hospital (4)	**Disciplines represented:** Aged care Chronic disease Executive services Health equity Mental health Occupational therapy Orthopaedics Pharmacy Physiotherapy Rehabilitation Renal Speech Pathology	**Staff role (*n*):** Nurse Navigator (1) Clinical Nurse Consultant (4) Nurse Unit Manager (1) Assistant Director (1) Administrator (1) Allied Health Professional (6)

#### 3.1.1 Summary of Main Findings

##### 3.1.1.1 ‘Don't Assume, Ask’

The overarching finding was that CALD participants (Workshop 1) felt they were not routinely offered the choice for telehealth due to staff assumptions about their low digital literacy, technological capability, and English proficiency. Despite this, they maintained a willingness to experience telehealth benefits such as avoiding travel, and a desire to have the choice with care modality.

Half of all health service staff participants (Workshops 2 and 3) said they were less likely to offer telehealth appointments to CALD consumers. Reasons they cited included: the perception that telehealth affects engagement and rapport; technical challenges around including interpreters on the video consultation; concern the client has poor digital literacy; language barrier in terms of how to communicate the process, and; overall cultural needs. These contributed to assumptions when making judgements on telehealth suitability for these consumers. Further, one health service staff participant perceived an inequity of telehealth access is linked to broader healthcare inequity issues:

“Even with existing healthcare access there are inequities that have not been addressed yet. So when you add the other layer of telehealth…it exacerbates the situation… It's not realistic to expect equitable telehealth delivery.”

– Staff member, Workshop 2

Interpreters (Workshop 1) often described themselves as “communication experts” but felt their skills were undervalued and at times, underutilised by clinicians and the health service. In Workshop 1, an interpreter participant commented, “*There's nothing humans have invented that is more sophisticated than language* –” and a consumer participant added, “– *and telehealth is a whole new language*.” Health service staff (Workshops 2 and 3) lacked education regarding how to book a video interpreter (often preferencing telephone only) and thought health executives should reinforce the organisation's position on telehealth and upskill consumers and staff.

This overarching theme highlighted the need for more education and reinforcement to promote clear processes for booking telehealth appointments for CALD consumers requiring an interpreter. These processes were displayed into an educational infographic with prompts to support clinician decision making before and during a telehealth appointment: questions to ask consumers, the advantages of seeing the patient and interpreter on video consultations instead of using telephone, and how staff can upskill on video interpreter use. Even if a clinician feels telephone is appropriate, the need to ask consumers their preference is highlighted by this participant quote:

“Telephone doesn't really work in our language, because our community, we talk with our eyes, we talk with our body language, we talk with our facial expressions, the nod of the head and the slight turn of the eyeball. It doesn't really work.”

–Consumer, Workshop 1

Each stakeholder was involved in the review and finalisation of the educational infographic and a high‐resolution version can be accessed and downloaded online [16].

#### 3.1.2 Recommendations to Enhance Telehealth Among CALD Communities

Originally it was expected that the different stakeholders would share similar experiences, solutions, and recommendations. However, the telehealth access gap in CALD groups was found to be much more complex, and the workshops identified a diversity of opinions and suggested actions to be taken. Furthermore, these actions targeted a multifaceted range of actors in the health system. The key messages to enhance telehealth use among CALD communities provided by each stakeholder group are summarised in Table 2. This included consumer recommendations, which focussed on generating awareness, self‐advocacy, preparation, and feedback on telehealth services. Clinician recommendations centred on the central overarching theme previously detailed ‘Don't assume, ask’, and expands this consideration to before, during and after the telehealth consultation. Interpreter findings focussed on encouraging themselves to drive consumer trust and confidence in telehealth, preparing consumers and conducting pre‐appointment engagement activities, and informing the health professional of consumers' concerns and requirements. For example, one consumer participant asked:

“In person, I get to have a chat with the interpreter in waiting room. Why can't we do that with telehealth?”

– Consumer, Workshop 1

**Table 2:** Recommendations for key stakeholder groups to enhance telehealth use among culturally and linguistically diverse (CALD) communities.

Group	Example messages to support telehealth promotion in CALD communities
**Consumers**	Increase awareness of telehealth benefits and suitability ○ Example statement could be: “Telehealth lets you receive care at home and choose how your care is provided. Some aspects of care (e.g., physical exam) may be best in‐person – discuss with your health provider how it could work for you.” Encourage self‐advocacy ○ Example statement could be: “Ask for telehealth when you would prefer it. You can also ask for an interpreter to join the call.” Prepare consumers for success ○ Example statement could be: “Prepare for your telehealth session ahead of time. Write down any questions, have medications on hand and any equipment you may need.” Encourage feedback ○ Example statement could be: “It is good to give feedback on the different ways your healthcare appointments happen (by phone, video, or in person). Your voice can advocate for the best possible care options.”
**Clinicians**	Ask rather than assume – Would consumers like the next appointment to be in person or via telehealth? Would they like an interpreter present? If so, do they have preference for male or female? Check – Do consumers know what telehealth is & how it could help them? Do they have the right equipment? Do they need additional support? Check your local processes for booking interpreters over telehealth. Set up for success – 1. Show the technology; 2. Explain how telehealth will work; 3. Practice ahead of time; 4. Have a backup plan. During the appointment – Say less and show more. Use pictures and gestures. Work with interpreters and acknowledge their skills. Allow extra time.
**Interpreters**	Help drive consumer confidence in telehealth – Encourage and educate consumers on using telehealth as an option. Prepare consumers – Use in‐person time to prepare consumers for a telehealth appointment, use demonstration, assess their capability and determine their need for support. Establish relationships – Utilise virtual waiting rooms and time pre‐appointments to build rapport. Etiquette – Be in a quiet, private space with appropriate lighting, high‐quality sound, and visuals. Inform the health professional if you do not think the consumer understands the information conveyed and/or if you would like to debrief before or after the session.
**Executives**	Promote telehealth interpreter use ○ For example you can circulate banners on computer screen savers or on staff intranet/website such as “You can book telehealth with interpreters!” Provide appropriate information – Translate telehealth instructions into multiple languages. Educate – Increase staff knowledge of interpreter role, train staff to conduct telehealth appointments with interpreters, use visual displays of telehealth with CALD consumers, and encourage interpreters to champion telehealth. Update existing models of care so guidelines and processes are in place with roles/responsibilities clear.

Finally, findings related specifically to health service executives centred on promoting telehealth interpreter use through education, training, policy, promotion, and information available in multiple languages.

## 4 Discussion

The exponential growth in telehealth uptake has transformed service delivery, offering convenience and accessibility to many individuals [17, 18]. However, recent data shows these benefits are less likely to reach people from CALD populations [7, 19]. Our current study attempts to explore this equity gap directly at the tertiary and community health service level. CALD consumers in our study reported wanting to be offered choice in care modality but reported they were not routinely offered telehealth. Health service staff participants reported being less likely to offer telehealth to CALD consumers due to perceived challenges with building rapport, technical challenges with including interpreters, perceptions around consumer digital health literacy and language barriers, and the complexity of setting up and facilitating telehealth appointments. Interpreters feel their skills are undervalued. To overcome these challenges, we developed a range of proactive strategies targeted to each of the key stakeholder groups involved in the study.

Clinician assumptions are a key driver as to whether telehealth is or is not offered [20, 21]. As discussed by Cook et al., one aspect of this gap is not so much inability to use telehealth but rather a digital exclusion based on preconceived frames of reference of health service staff [20]. Beyond the fact that choice in the modality of care is not consistently offered in Australia [22], the need to include a third party, an interpreter service, in a telehealth appointment might add another layer of complexity. However, CALD consumers can be supported to use telehealth [23, 24, 25]. For example, previous literature has suggested healthcare organisations purchase and provide consumers with Wi‐Fi hotspots and mobile devices, hire digital navigator roles and culturally competent health workers, advocate for digital inclusion policies, or translate telehealth resources into different languages [23, 24, 25]. Health technology experts and equity researchers agree that community‐based initiatives to promote digital literacy may be more sustainable than individually run telehealth training programs and that systems need to adapt to different levels of digital literacy [25]. Ongoing evaluation of services and consumer experiences is also important to identify areas for improvement [23]. Further, to support consumers to access telehealth, staff need to feel confident and capable of delivering their care remotely and working with an interpreter. Hence, training is required and should include building rapport remotely with CALD populations and working with interpreters [24]. Clear procedures for booking and using interpreter services online must also be communicated and promoted.

Accessing interpreter services in a timely manner is a challenge across many health services [26, 27, 28]. Digital health offers excellent opportunities to connect and share skilled interpreters across jurisdictional boundaries. Language service apps (e.g. 2M lingo, Care to Translate, etc.) can enable health professionals to add an on‐demand interpreter to their current video call. Healthcare providers in Australia, such as the Royal Melbourne Hospital, have enabled video remote interpreting and reported that it has facilitated timely access to interpreters and overcomes geographic and language barriers [29].

Innovations in telehealth interpreting services are reducing costs while delivering high‐quality professional interpreter services, ensuring the best overall care for those in need [30]. For example, video‐interpreters on demand (which enable remote connection to interpreters based in a wide range of locations) are being used privately and in other health settings internationally [31, 32]. While such services have potential to increase timely access to interpreters, they appear to be under‐utilised, at least in the health services studied presently. Hence, making such services widely accessible remains an important challenge to address [30].

Building trust in digital health technologies, including telehealth, is necessary to close access gaps. Trust in digital health from the consumer perspective is associated with their level of digital literacy and positive experiences with previous telehealth appointments [33]. Trust can predict consumer acceptance of certain digital health modalities, and it is positively associated with intention to use telehealth [8, 34, 35, 36, 37]. Therefore, education and support are key on the consumer end.

Our consumers highlighted the need to raise awareness of telehealth among CALD groups to encourage self‐advocacy. Initiatives should be implemented to enhance digital literacy and support consumers in confidently navigating telehealth platforms. Australia's National Digital Health Strategy prioritises support for consumer digital health literacy, and initiatives are underway to codesign and deliver programs [38]. One such initiative being trialled by some health services is the use of a digital health navigator to support both consumers and clinicians in accessing and using telehealth more effectively [39].

Our study findings converge with academic literature addressing the digital divide. Closing the digital divide requires a multi‐level approach, and various stakeholders need to undertake strategies to tackle the multiple dimensions of telehealth access. These strategies include describing and measuring the extent of the digital divide in their context; promoting digital literacy of consumers; supporting shared decision‐making and personalised approaches; and capacity building of health service providers ensuring quality, cultural safety and ethical service provision.

This study has important limitations to consider. Firstly, our CALD consumers had very limited experience with telehealth which highlights the difficulty in designing specific solutions for this group of consumers. Where there was some exposure, it was with a telephone modality. Similarly, our clinicians had experience with telehealth interpreter services but with a diverse set of processes, opinions and expectations, which made synthesizing these difficult. Also, the limited number of languages and cultures explored in this study might not be representative of the multicultural myriads of people living in Australia. This meant that thematic saturation was not appropriate, however, our small sample had sufficient information power for the research question and hypothesis generating purposes of the study. The generalisability of the findings may be limited to the services and populations with limited telehealth experience and CALD demographic who participated. However, drawing on the current findings, the barrier caused by the need to include an interpreter is most likely an issue regardless of language type. The main strength is that the data was collected from a range of stakeholders shedding light on this important area of inequity. A further strength is the solutions generated by stakeholders affected by this health service delivery. Finally, having two CALD clinician‐researchers involved in this research provided a unique insider perspective potentially strengthening the analysis and results [40].

## 5 Conclusion

While the exponential growth of telehealth has many benefits for consumers and the healthcare sector, addressing health equity concerns accompanying its implementation is essential. This study demonstrates that providing CALD consumers with choice on their care modality, without prejudice, is paramount. By developing strategies to bridge the gap between those who can access telehealth and those who cannot, we can increase the likelihood that telehealth works as a tool for equitable health care delivery. Action is required to improve awareness, connectivity, capability, trust and willingness to engage in telehealth, promoting a healthcare system that benefits the entire Australian community, despite cultural and language diversity. Our suggestions and recommendations are an important step in improving equity of access to health services, including telehealth.

## Author Contributions

**Jaimon T Kelly:** methodology, data curation, writing–original draft, writing–review and editing, formal analysis, supervision. **Monica L Taylor:** project administration, data curation, writing–original draft, writing–review and editing, formal analysis. **Victor M Gallegos‐Rejas:** writing–original draft, writing–review and editing. **Susan Pager:** conceptualization, methodology, resources, funding acquisition, writing–review and editing, formal analysis, data curation. **Soraia de Camargo Catapan:** writing–review and editing. **Karen Lucas:** conceptualization, methodology, data curation, formal analysis, writing–review and editing, funding acquisition, resources. **Angel Bogicevic:** conceptualization, methodology, data curation, resources, writing–review and editing. **Anthony C Smith:** formal analysis, resources, writing–review and editing. **Centaine Snoswell:** writing–review and editing. **Helen M Haydon:** writing–review and editing. **Emma E Thomas:** conceptualization, methodology, writing–original draft, writing–review and editing, supervision, funding acquisition, resources.

## Ethics Statement

This project was approved by Metro South Health and The University of Queensland's Human Research Ethics Committees (HREC/2021/QMS/81523 and 2022/HE001211).

## Conflicts of Interest

The authors declare no conflicts of interest.

## Data Availability

The data that support the findings of this study are available from the corresponding author upon reasonable request.
